# A Wage Limit for In-Patients

**Published:** 1890-06-21

**Authors:** 


### A WAGE LIMIT FOR IN-PATIENTS.

Dr. R. R. Rentoul, of Liverpool, writes:—Will you or any of the readers of your journal let me know if they are aware of the existence of a rule referring to the "wage limit" which debars applicants from receiving in-patient medical relief at the voluntary hospitals? I do not refer to the "wage limit" for out-patients.

